# Derivatives and intermediates of betulonic and platanic acid as effective antibacterial agents

**DOI:** 10.1007/s12223-026-01455-9

**Published:** 2026-04-16

**Authors:** Lucie Černá, Angelina Massyagutova, Uladzimir Bildziukevich, Zdeněk Wimmer, Petra Lovecká

**Affiliations:** 1 Department of Biochemistry and Microbiology, University of Chemistry and Technology in Prague, Technická 5, Prague 6, 16628 Czech Republic; 2 Isotope Laboratory, Institute of Experimental Botany of the Czech Academy of Sciences, Vídeňská 1083, Prague 4, 14220 Czech Republic; 3 Department of Chemistry of Natural Compounds, University of Chemistry and Technology in Prague, Technická 5, Prague 6, 16628 Czech Republic

**Keywords:** Antimicrobial activity, Oxime derivatives, Betulonic acid, Platanic acid

## Abstract

Triterpenoid acids are secondary metabolites known to possess various beneficial biological activities. Their synthetic derivatives often display improved pharmacological properties and higher levels of efficacy. In this study, we investigated, the antibacterial effects of 13 synthesized oxime derivatives and 4 intermediates of betulonic and platanic acid, comparing them with those of the unmodified forms. Six Gram-positive pathogenic human bacteria, including four resistant strains from the genus *Staphylococcus*, were selected for testing together with two Gram-negative bacteria. All combinations of the prepared bacterial suspensions and 17 samples (500 − 62.5 µmol/L) were placed on 96-well microtitration plates. The microdilution method was used to measure the absorbance of the samples, from which bacterial growth curves were derived. Resazurin tests were performed to obtain fluorescence values and, thereby, determine the metabolic activity and bactericidal effects. The derivatives and intermediates exhibited higher sensitivity to the Gram-positive bacteria. The most effective samples, for which 90–100% growth inhibition was observed against resistant strains (*S. aureus* 368, *S.* *epidermidis* 335), were the oxime ester derivative of betulonic acid synthesized from 1-adamantylacetic acid (**14**; 500 µmol/L) and the C-3 acetate intermediate of platanic acid (**18**; 500 µmol/L). Sample **18** also showed 90–100% inhibition against *S. aureus* 670. Thus, our results suggest that the tested triterpenoid derivatives and intermediates have considerable potential for the development of new antibiotic therapies.

**Supplementary Information:**

The online version contains supplementary material available at https://doi.org/10.1007/s12223-026-01455-9.

## Introduction

The rapid spread of resistant bacterial strains has led to an urgent search for new antibiotics (Karaman et al. 2020; Davies et al. 2010). WHO has declared antibiotic resistance to be one of the three major threats to public health and published a list of pathogenic bacteria that are moderately, highly or critically resistant to current treatments (Karaman et al. 2020; Breijyeh et al. 2020). Furthermore, many pathogenic strains have evolved into multidrug-resistant (MDR) forms capable of resisting multiple classes of antibiotics simultaneously (Davies and Davies 2010). For example, certain strains of *Staphylococcus aureus*, particularly methicillin-resistant *S. aureus* (MRSA), have been reported to be resistant to all *β*-lactam antibiotics and to up to 20 different antimicrobial compounds, representing most of the available antimicrobial classes (Chung 2020). Current approaches to the discovery of new antibacterial agents involve various strategies; the most common being (i) the development of compounds that inhibit resistance mechanisms, such as *β*-lactamase inhibitors (e.g. avibactam) or efflux pump inhibitors, and (ii) the synthesis of derivatives that modify the activity of known antibacterial structures, such as tigecycline or the siderophore-linked cephalosporin cefiderocol, in order to circumvent such mechanisms. More recent strategies comprise phage therapy, the use of nanoparticles, the development of inhibitors of virulence factors or the design of antibacterially effective peptides and proteins (Vila et al. 2020).

Aside from these approaches, another approach can focus on natural products and the return of natural product screening to discover new therapeutics, highlighting the development of naturally derived or inspired compounds with antibacterial activity (Breijyeh et al. 2020). Plant-derived secondary metabolites are a major source of bioactive compounds with potential efficacy in the treatment of bacterial infections. Of these, the class of triterpenoids is of particular interest because they exhibit a wide range of biological activities and are known for their significant pharmacological effects. For example, they have been used to reduce blood glucose levels and blood pressure. In addition, their anti-inflammatory and antioxidant effects can help protect the liver (Li et al. 2023; Khwaza et al. 2021). Moreover, they have demonstrated antimicrobial, antiviral and anticancer activities. Triterpenoids occur in nature in diverse structures, including in the form of tetra- or pentacyclic structures (Karaman et al. 2020; Martinez 2014).

Pentacyclic triterpenoids are derived from the isoprenoid biosynthetic pathway. This pathway involves the cyclization of oxidosqualene and six isoprene units, meaning that the basic structure of pentacyclic triterpenoids contains 30 carbon atoms (Li et al. 2023). They are divided into three classes whose structure is derived from ursane, lupane or oleanane (Chung 2020). Currently, research is primarily focused on lupane-type triterpenoids, specifically on lupeol, platanic acid, betulin, and betulinic and betulonic acids. Lupane pentacyclic triterpenoids have four six-membered rings and one five-membered ring (the E ring) in which the isoprenyl group at C-19 is in the *α*-configuration (Li et al. 2023). The antimicrobial activity of betulonic and platanic acids has been demonstrated against Gram-positive bacteria, with greatest sensitivity demonstrated against *S. aureus* and *Enterococcus faecalis* (Haque et al. 2014). Kazakova et al. (2010) showed that betulonic acid concentrations of up to 80 mg/mL reduced the growth of *S. aureus*, and that concentrations of 90 and 100 mg/mL completely suppressed it. However, for Gram-negative *Klebsiella pneumonia*, its growth was reduced but not completely suppressed (Kazakova et al. 2010).

Because pentacyclic triterpenoids have various limitations - such as low water solubility, low selectivity, poor bioavailability, and short half-life, that currently prevent their clinical application, structural modifications have been proposed to enhance their therapeutic potential. Most such studies have focused on the preparation and evaluation the biological activity of amide or amine derivatives of betulonic and platanic acids (Khwaza et al. 2021; Lombrea et al. 2021). Bednarczyk-Cwynar et al. (2015) reviewed the synthesis of oxime and oxime ester derivatives of triterpenoid acids. Subsequently, Kahnt et al. (2018), Kozubek et al. (2022) and Lombrea et al. (2021) all reported promising results with the use of such derivatives. In our previous study, oxime synthesis was employed to successfully prepare completely new derivatives incorporating nitrogen in the structure (Černá et al. 2024). This approach appears to be effective, but the antibacterial properties of such derivatives has not yet been fully explored, especially for platanic acid derivatives.

In this study, we evaluated the antibacterial effectiveness of a new series of 13 synthesized oxime derivatives, some of which contain a nitrogen heterocycle, and 4 intermediates of betulonic and platanic acids. Using disk diffusion, spectrophotometric microdilution assay and the resazurin assay, the bacteriostatic and bactericidal properties of the substances are determined against four model Gram-positive and Gram-negative bacteria: *S. aureus* and *E. faecalis*; *Escherichia coli* and *Pseudomonas aeruginosa*. All results are compared with those obtained for unmodified forms of betulonic and platanic acids. Because the derivatives and intermediates exhibitited higher sensitivity to the Gram-positive bacteria, the effects are more closely determined for the resistant clinical isolates *S. epidermidis* 335, *S. hominis* 334, *S. aureus* 368 and *S. aureus* 670. Overall, our results suggest that some of the oxime-based derivatives and intermediates have considerable potential for the development of new antibacterial therapies.

## Materials and methods

### Chemicals and laboratory instruments

Chemicals Mueller-Hinton Broth (MHB) and Mueller-Hinton Agar (MHA) were purchased from Oxoid (United Kingdom); resazurin powder from HiMedia Laboratories (India); antibiotics: vancomycin hydrochloride from HiMedia Laboratories and kanamycin sulfate from Sigma-Aldrich (USA). The solvent dimethyl sulfoxide (DMSO) was from Lach-Ner (Czech Republic). Laboratory instruments used included: Class II Biohazard laminar flow cabinet-Clean Air (United Kingdom); Incubation chamber-Memmert GmbH & Co. KG (Germany); Synergy spectrophotometer-BioTek (USA); Vortex mixer-Velp Scientifica (Italy); Densilameter II-Erba Lachema; Digital analytical balance ER 120 A-Sartorius; Digital balance PM 400-Mettler; Autoclave 3870 TUTTNAUER-Tuttnauer.

### Preparation of solutions and culture media

MHB: 22 g of powder per 1000 mL distilled H_2_O; MHA: 38 g of powder in 1000 mL distilled H_2_O. Resazurin solution: a stock solution of a concentration of 0.01 mg/mL was prepared. Antibiotic solutions (positive control): a stock solution of 10 mg/mL was used; vancomycin for Gram-positive bacteria, kanamycin for Gram-negative.

### Overview of microorganisms used

Czech Collection of Microorganisms - *S. aureus* CCM 4516; *E. faecalis* CCM 4224; *E. coli* CCM 4517; *P. aeruginosa* CCM 1961; Clinical isolates obtained in cooperation with the University hospital of Královské Vinohrady (Czech Republic, Prague 10) - *S. epidermidis* 335 M0000335 C0719102; *S. hominis* 334 M0000334 C0719103; *S. aureus* 368 M0000368 G0278785; *S. aureus* 670 M0000371 F0313670. The clinical and microbiological characteristics of all tested strains are summarized in (Table 1).

**Table 1:** General characteristics of tested strains (Hall et al. 2017; Manandhar et al. 2025; Mousavi et al. 2018; Qureshi et al. 2025; Sharma et al. 2023; Venkatesan et al. 2015)

Species	Strain ID	Type	Clinical-microbial properties
*S. aureus*	CCM 4516	Reference strain	Often methicillin-resistant; moderate to strong biofilm formation; high virulence, toxin production
*E. faecalis*	CCM 4224	Reference strain	Often vancomycin-resistant; opportunistic pathogen; urinary tract infection and endocarditis
*E. coli*	CCM 4517	Reference strain	Often MDR; uropathogenic and gastrointestinal infections
*P. aeruginosa*	CCM 1961	Reference strain	Intrinsic resistance to many antibiotics; MDR possible; strong biofilm formation; opportunistic pathogen; biofilm-associated chronic infections (chronic lung infections in cystic fibrosis patients)
*S. epidermidis* 335	M0000335 C0719102	Clinical isolate	Often MDR; strong biofilm formation; opportunistic pathogen; device-associated infections; resistance to: OXA, ERY, CLI, CIP, TET and COT
*S. hominis* 334	M0000334 C0719103	Clinical isolate	Can be methicillin-resistant; moderate to strong biofilm formation; opportunistic pathogen; biofilm-mediated infections; resistance to: OXA, ERY, CLI, CIP, GEN, LNZ, CMP and COT
*S. aureus* 368	M0000368 G0278785	Clinical isolate	Often methicillin-resistant; moderate to strong biofilm formation; high virulence and toxin production; resistance to: OXA, ERY, CLI, CIP and GEN
*S. aureus* 670	M0000371 F0313670	Clinical isolate	Often methicillin-resistant; moderate to strong biofilm formation; high virulence and toxin production; resistance to: OXA and GEN

Abbreviations of antibiotics: CIP – ciprofloxacin, CLI – clindamycin, CMP – chloramphenicol, COT – cotrimoxazole, ERY – erythromycin, GEN – gentamicin, LNZ – linezolid, OXA – oxacillin and TET – tetracycline. Additional abbreviations: MDR – Multidrug-resistant

### Preparation of the microbial suspension and culture conditions

Petri dishes with MHA were inoculated with a stock culture of the bacterium and were subsequently incubated for 16 h at 37 °C, except for *E. faecalis*, which was incubated at 28 °C.

### Preparation of solutions of tested compounds

The synthesized compounds (Černá et al. 2024) were first weighed on an analytical balance and dissolved in 1 mL of DMSO to obtain 10 mmol/L solutions. Some samples were not completely dissolved after the addition of the solvent and were therefore placed in a sonication bath (for 1 h). The samples were then decimally diluted with MHB to form 1 mmol/L solutions. An overview of all tested compounds is summarized in Tables 2 and 3. Along with these samples, antibiotic solutions were also used in the testing as a positive control and a diluted DMSO solution as a negative control.

**Table 2:**
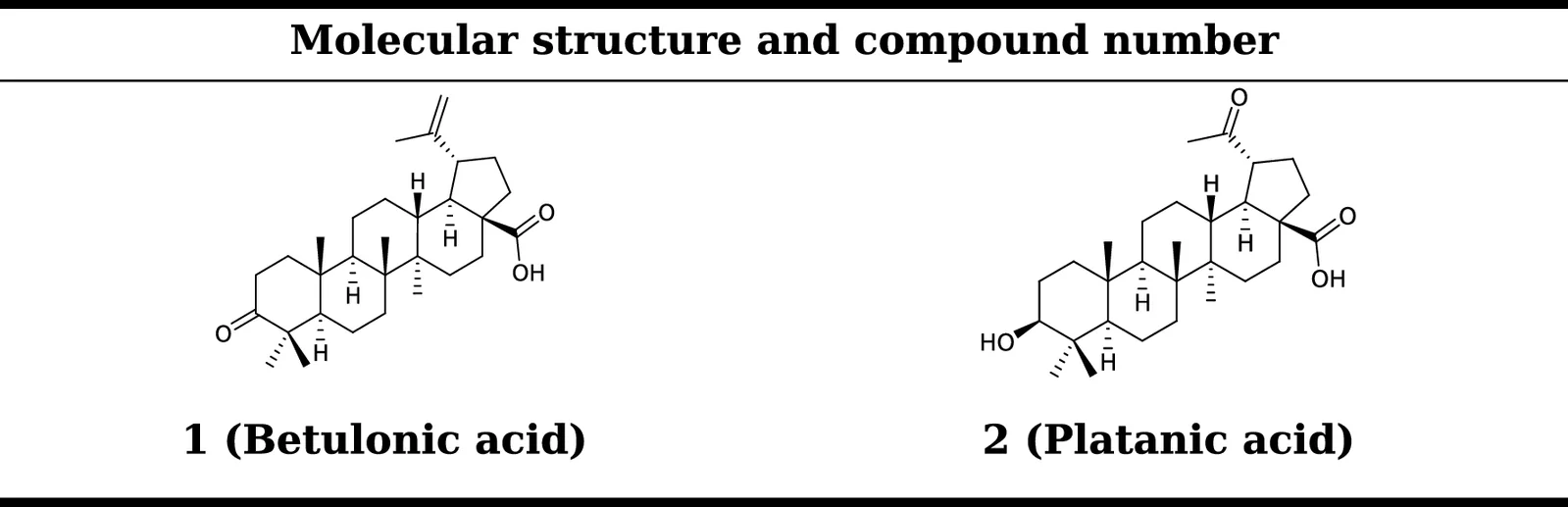
Structures of betulonic acid and platanic acid

**Table 3:**
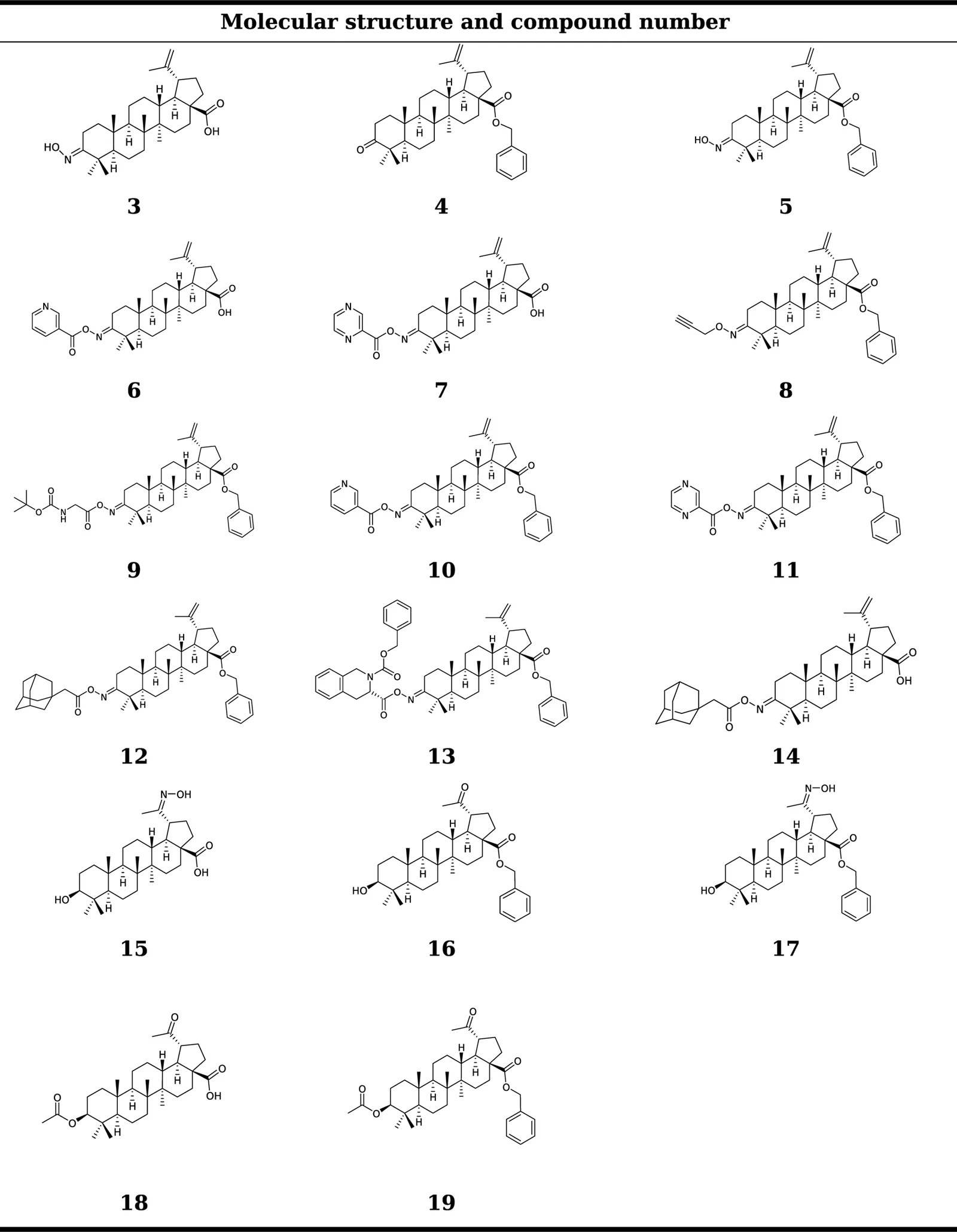
Overview of the tested triterpenoic acid derivatives

### Antibacterial assay

#### Disk diffusion method

The tested bacterial cultures were prepared by 16 h cultivation on MHA at 28–37 °C. Bacterial suspensions were prepared by resuspension in MHB and adjusted to the 0.5 McFarland standard. An aliquot of 100 µL of the suspension was evenly spread over the surface of MHA. Sterile paper disks were loaded with 20 µL of test solutions (1 mmol/L) and placed onto the agar surface. After 24 h incubation, inhibition zone diameters were measured and bacteriostatic activity was evaluated. Standard antibiotics, including vancomycin and kanamycin, were used as reference compounds. This method was performed according to CLSI guidelines (Institute 2023a) and served as a rapid screening assay complementing the quantitative microdilution method.

#### Microdilution method

96-well microtiter plates were used for the microdilution method. A concentration series of samples (500 − 62.5 mmol/L) was prepared in the plates by successive two-fold dilution and inoculated with two-fold diluted bacterial suspensions adjusted to 0.5 McFarland, resulting in a final concentration of 5 × 10^5^ CFU/mL. The final volume in each well was 200 µL, and all measurements were performed in triplicate. Bacterial growth was monitored for 24 h by measuring absorbance at 625 nm (A_625_) using a Synergy spectrophotometer at 28–35 °C. Growth curves were constructed, and percentage inhibition as well as the specific growth rate *µ* [1/h] were calculated (using Eq. 1). Standard antibiotics, including vancomycin and kanamycin, were used as reference compounds. The microdilution method was performed according to CLSI guidelines (Institute 2023b).

1 $$\mu=\frac{\log A_2-\log A_1}{t_2-t_1}$$

*A*_*1*_… the average value of the measured absorbance at the beginning of exponential growth, i.e. at time *t*_*1*_

*A*_*2*_… the average value of the measured absorbance at the end of exponential growth, i.e. at time *t*_*2*_

#### Resazurin assay

After 24 h cultivation of the 96-well microtiter plate used for the microdilution test, 100 µL were transferred to a new 96-well microtiter plate. 100 µL of resazurin solution was added to each well immediately afterwards. After approximately 30 min, the color change was quantified by measuring fluorescence using a Synergy spectrophotometer set to λ_excitation_ = 560 nm and λ_emission_ = 590 nm. From these measurements, the percentage of inhibition and the number of metabolically active cells were determined (using Eq. 2), allowing assessment of bactericidal activity.

2 $$\%inhibition=\left(\frac{\left(\Phi_{DMSO}-\Phi_{PM}\right)-\left(\Phi_S-\Phi_{PM}\right)}{\left(\Phi_{DMSO}-\Phi_{PM}\right)}\right)\cdot100$$

*Φ*_*S*_… average value of the measured fluorescence of the sample

*Φ*_*DMSO*_…average value of the measured fluorescence in the presence of DMSO

*Φ*_*PM*_…average value of the measured fluorescence of the pure medium MHB

### Statistical analysis

All experiments were conducted in technical triplicates and data were analyzed using MS Excel. Data for the growth curves and for tabulated results are presented as mean ± standard deviation (SD), with standard deviations calculated using the STDEV.S function in Excel to estimate variability based on sample data. Standard error was also estimated for each experimental point and is indicated in the figures as error bars.

## Results and discussion

### Results

The antibacterial activity was evaluated using three methods: (i) the disk diffusion method, (ii) the microdilution method, and (iii) the resazurin assay. Each compound was tested in a total of four concentrations (500, 250, 125 and 62.5 µmol/L). In the first step, a screening was performed on four model pathogenic bacteria: Gram-positive *S. aureus* and *E. faecalis*, and Gram-negative *P. aeruginosa* and *E. coli*. Preliminary results obtained from the disk diffusion method showed inhibition zones of approximately 15 to 29.9 mm in diameter for *S. aureus* with derivatives **6**, **14**, **15**, **16** and **19**, and for *E. faecalis*, with derivatives **8** and **16** (see Table 4, left columns).

**Table 4:** Antibacterial activity of the tested triterpenoid derivatives

Tested substance	In vitro antibacterial activity											
	*S. aureus*			*E. faecalis*			*E. coli*			*P. aeruginosa*		
	d [mm]	µ × 10^− 2^ [1/h]	I [%]	d [mm]	µ × 10^− 2^ [1/h]	I [%]	d [mm]	µ × 10^− 2^ [1/h]	I [%]	d [mm]	µ × 10^− 2^ [1/h]	I [%]
1% DMSO	-	4.09	0.0	-	3.35	0.0	-	6.06	0.0	-	7.70	0.0
Vancomycin	+++	**0.00**	**100.0**	+++	**0.00**	**100.0**	-	-	-	-	-	-
Kanamycin	-	-	-	-	-	-	+++	**0.00**	**100.0**	+++	**0.00**	**100.0**
**1**	+	**0.00**	-	-	**0.00**	**93.9**	-	5.59	23.2	-	1.82	-
**2**	-	**0.80**	-	-	1.67	5.4	-	7.53	2.6	-	3.67	-
**3**	-	**0.00**	-	+	**0.00**	**100.0**	-	5.59	6.0	-	2.54	-
**4**	-	**0.88**	-	-	1.43	**86.6**	-	3.94	-	-	3.19	-
**5**	-	1.98	13.6	-	1.75	55.7	-	7.05	0.9	-	3.64	-
**6**	++	2.29	-	-	**0.00**	19.0	-	2.94	-	-	3.63	-
**7**	+	**0.00**	3.2	-	**0.00**	60.4	-	2.97	-	-	3.12	-
**8**	-	**0.96**	10.5	++	**0.75**	61.0	-	2.78	0.9	-	2.14	-
**9**	-	2.33	-	-	6.61	-	-	5.11	-	-	3.46	22.8
**10**	-	7.17	-	-	9.45	-	-	3.78	-	-	4.00	-
**11**	-	5.45	12.1	-	2.82	6.5	-	1.77	-	-	4.88	-
**12**	+	1.34	36.9	-	1.28	18.8	-	2.23	16.7	-	5.89	-
**13**	-	2.79	-	-	4.93	12.6	-	4.29	-	-	2.61	16.6
**14**	++	**0.00**	64.6	-	**0.00**	70.5	+	3.63	-	-	3.02	32.6
**15**	++	2.28	-	-	1.01	9.9	-	5.54	-	-	5.15	-
**16**	++	1.12	-	++	1.07	69.3	-	4.12	-	-	2.30	-
**17**	-	**0.97**	43.0	-	1.50	74.5	-	5.43	13.0	-	5.21	-
**18**	+	**0.00**	**96.2**	+	4.03	**83.0**	-	12.98	30.7	-	3.14	24.4
**19**	++	**0.93**	-	-	1.49	23.3	++	6.03	4.1	-	3.93	-

The table summarizes the antibacterial activity after exposure to the samples against *S. aureus*, *E. faecalis*, *E. coli*, and *P. aeruginosa* evaluated using three complementary assays: (1) The disk diffusion method (after 24 h; 10 mM, 37 °C or 28 °C), expressed as the average inhibition zone diameter *d* [mm], where “+++” indicates more than 30 mm, “++” indicates diameter interval 15 to 29.9 mm, “+” indicates diameter interval 10.1 to 14.9 mm, and “-” indicates no activity; (2) Specific growth rates *µ* × 10^− 2^ [1/h] determined using the microdilution method (after 24 h, 500 µmol/L, 35 °C or 28 °C) All values are reported after scaling by a factor of 10^2^ to improve clarity. The actual value of *µ* is obtained by multiplying the values reported in the table by 10^− 2^. (3) Inhibition of metabolic activity in % obtained from the cell viability test - resazurin assay (after 24 h, 500 µmol/L, 35 °C or 28 °C). The symbol “-” indicates the presence of metabolic activity, corresponding to negative growth inhibition (i.e., no inhibition), meaning that the bacteria grew at or above the level of the negative control. Numeric values represent the percentage of growth inhibition (100% = complete inhibition). The measurement uncertainty (± 15%) for the data of specific growth rates and inhibition of metabolic activity is provided in the Supplementary material

**Fig. 1:**
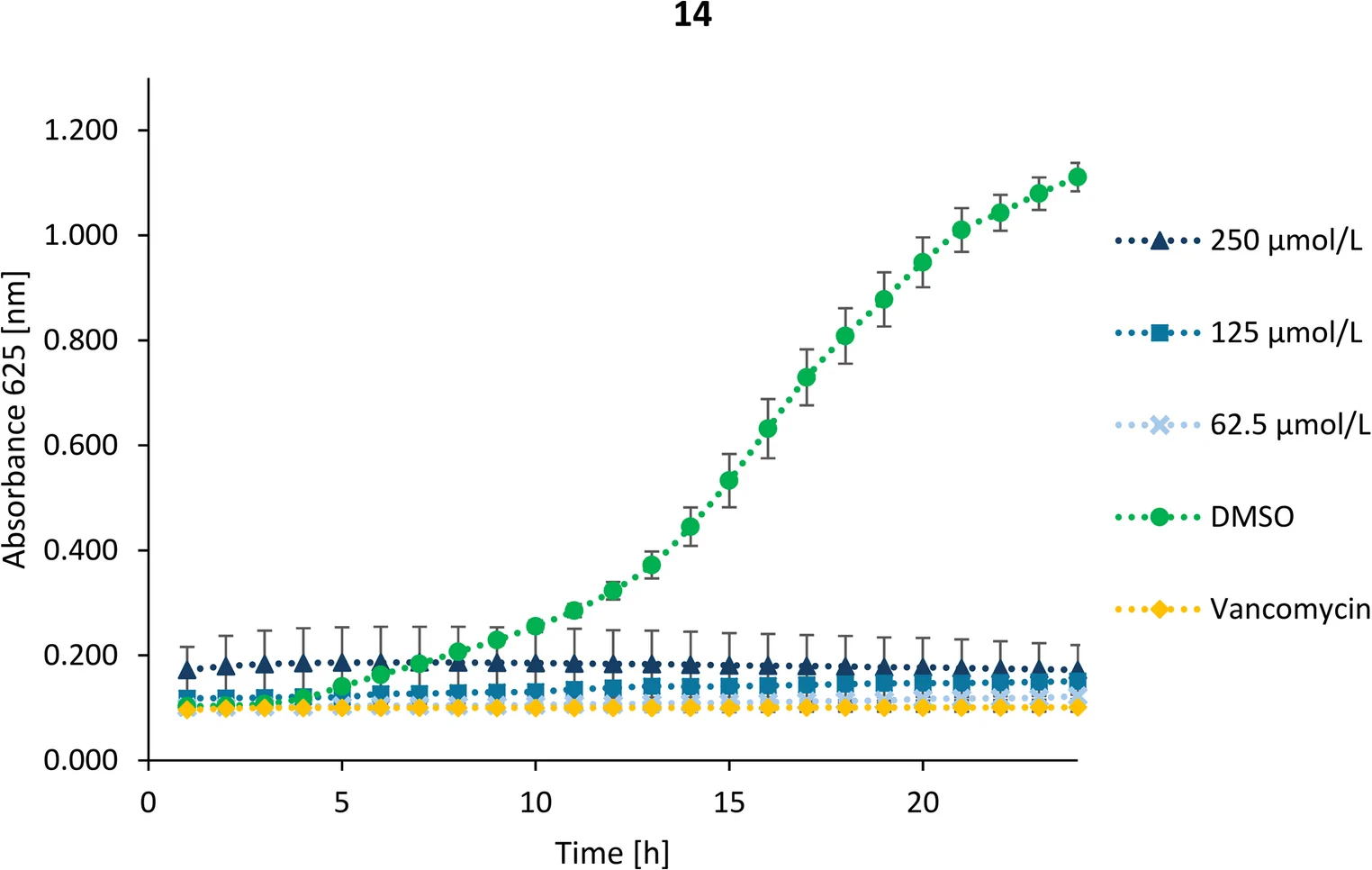
Growth curves of *S. aureus* after exposure to derivative **14** at concentrations of 250 µmol/L, 125 µmol/L and 62.5 µmol/L and incubation at 35 °C for 24 h. The 1% DMSO sample represents the bacterial growth curve as a negative control. The positive control is the bacterial growth curve inhibited by vancomycin at the concentration of 250 µg/mL

Subsequently, the derivatives were evaluated using the microdilution method (see Table 4). Most samples showed antibacterial activity predominantly against Gram-positive strains. Inhibition of growth was observed mainly for *S. aureus* and *E. faecalis* across the majority of the derivatives measured as the specific growth rate *µ* [1/h]. Very strong antibacterial effects of the oxime ester derivative of betulonic acid substituted by the 1-adamantyl acetic acid skeleton (**14**) against *S. aureus* were observed as 100% inhibition of growth occurred even at the lowest tested concentration of 62.5 µmol/L (Fig. 1). The same derivative **14** also showed significant effects against the growth of *E. faecalis* (500 µmol/L; Table 4). Similarly, substances **3**, **6** and **7** exhibited antibacterial activity against the growth of *E. faecalis*. However, this antibacterial effect was also observed with the reference unmodified betulonic acid (**1**). Approximately 100% inhibition of *S. aureus* growth even in lower concentration (≤ 125 µmol/L) occurred with unmodified betulonic acid (**1**), the oxime derivative of betulonic acid (**3**), the oxime ester derivative of betulonic acid substituted by the pyrazine-2-carboxylic acid (**7**) and the acetate intermediate of platanic acid (**18**) (see **Supplementary material**).

Some of these derivatives showed complete inhibition of growth, and their specific growth rate was calculated as *µ* = 0.00 × 10^− 2^ [1/h] for the concentration of 500 µmol/L for derivatives **3**, **7**, **14** and **18** against *S. aureus* and **3**, **6**, **7** and **14** against *E. faecalis*. The zero value of the specific growth rate determined against both representatives of Gram-positive bacteria occurred for **3**, **7** and **14** (Table 4). Examination of the growth curves provided in the Supplementary material allowed the estimation of the minimum inhibitory concentration (MIC) for the most active derivatives, which in most cases corresponded to the concentration of 125 µmol/L.

The resazurin test proved inhibition of metabolic activity *I* [%], and so bactericidal effects, of betulonic acid (**1**) and tested derivatives **3**, **4** and **18** with inhibition ranging between 80 and 100% against *E. faecalis*. The intermediate **18** also showed the inhibition of 90% against *S. aureus* (see Table 4). These results indicate that most substances had only bacteriostatic effects on the bacterial cell.

**Fig. 2:**
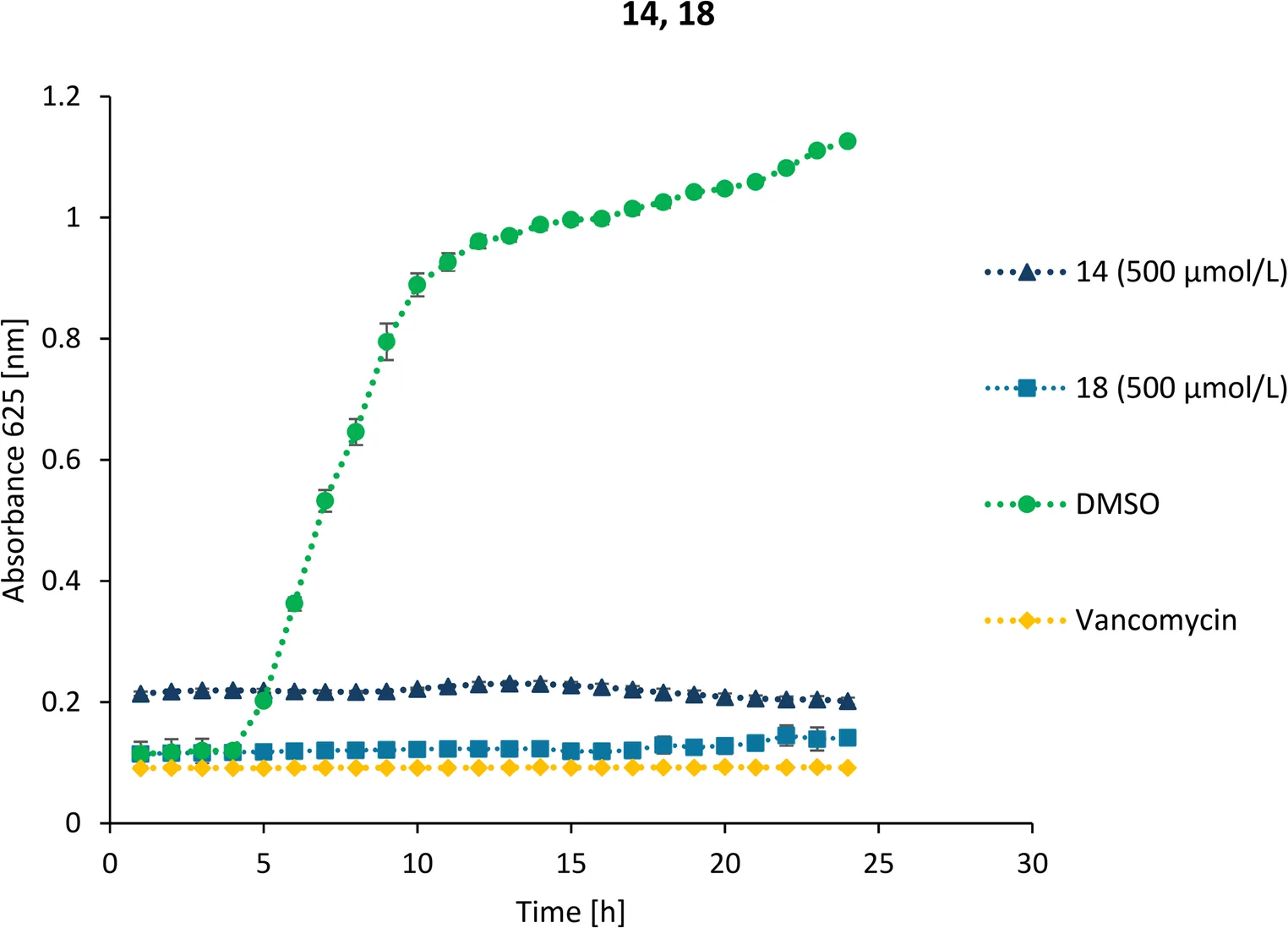
Growth curves of resistant *S. aureus* 368 after exposure to derivatives **14** or **18** at the concentration of 500 µmol/L and incubation at 35 °C for 24 h. The 5% DMSO sample represents the bacterial growth curve as a negative control. The positive control is the bacterial growth curve inhibited by vancomycin at a concentration of 250 µg/mL

Due to the significant sensitivity observed against *S. aureus*, selected compounds (**1**, **2**, **3**, **7**, **14**, **17** and **18**) were also tested against resistant clinical isolates obtained from the hospital environment. Specifically, the isolates were *S. aureus* 670, *S. aureus* 368, *S. hominis* 334 and *S. epidermidis* 335. Antibacterial properties at the concentration of 500 µmol/L were found mainly against *S. aureus* 368 and *S. epidermidis* 335, and also against *S. aureus* 670 for **2**, **3**, **14**, **17** and **18**. However out of the derivatives, only **14** and **18** achieved significant inhibition of metabolic activity ranging around 100% (Table 5; Fig. 2).

**Table 5:** Inhibition of metabolic activity in % obtained from the cell viability test (resazurin assay) in *S. aureus* 670, *S. aureus* 368, *S. hominis* 334 and *S. epidermidis* 335 after exposure to the samples at a concentration of 500 µmol/L following 23 h of incubation at 35 °C

Tested substance	Inhibition of metabolic activity [%]			
	*S. aureus* 670	*S. aureus* 368	*S. hominis* 334	*S. epidermidis* 335
5% DMSO	0.0 ± 0.00	0.0 ± 0.00	0.0 ± 0.00	0.0 ± 0.00
Vancomycin	**100.0 ± 15.00**	**100.0 ± 15.00**	**100.0 ± 15.00**	**100.0 ± 15.00**
**1**	14.7 ± 2.21	68.7 ± 10.31	-	**100.0 ± 15.00**
**2**	67.2 ± 10.08	**100.0 ± 15.00**	-	-
**3**	-	-	72.1 ± 10.82	**94.8 ± 14.22**
**7**	8.3 ± 1.25	53.8 ± 8.07	-	5.5 ± 0.83
**14**	56.9 ± 8.54	**94.4 ± 14.16**	53.8 ± 8.07	**99.4 ± 14.91**
**17**	73.7 ± 11.06	**89.8 ± 13.47**	-	1.2 ± 0.18
**18**	**97.1 ± 14.57**	**100.0 ± 15.00**	12.6 ± 1.89	**100.0 ± 15.00**

The results are subject to a ± 15% error. The symbol “-“ indicates the presence of metabolic activity, corresponding to negative growth inhibition (i.e., no inhibition), meaning that the bacteria grew at or above the level of the negative control. Numeric values represent the percentage of growth inhibition (100% = complete inhibition)

Among the structural modifications of platanic acid, the acetate intermediate (**18**) exhibited the most promising biological activity. In the case of betulonic acid, the highest efficacy was observed particularly in its oxime derivatives, including the simple oxime (**3**) and two oxime ester derivatives, which were synthesized using pyrazine-2-carboxylic acid (**7**) and 1-adamantylacetic acid (**14**), respectively.

### Discussion

Bacterial infections remain a major global healthcare concern and the development of novel antibacterial agents continues to be a critical area of research (Haque et al. 2014; Lombrea et al. 2021). Reports by Kazakova et al. (2010) have highlighted triterpenoids as promising candidates in the search for new antibacterial agents thanks to their high efficiency in treating infections. Based on these findings, our research focuses on the investigation of triterpenoid compounds (betulonic acid and platanic acid) with potential therapeutic activity against bacterial infections. Another reason for their selection was the fact that both these triterpenoic acids have not yet been fully explored in terms of their biological activity and potential therapeutic applications. On the other hand, they exhibit a range of well-known beneficial properties in the medicinal field. According to the study by Kahnt et al. (2018), promising results were observed for derivatives of platanic acid after the incorporation of functional groups containing nitrogen atoms.

Therefore, our study focused on testing derivatives containing an oxime group and evaluating whether the modification of triterpenoic acid can lead to improved antibacterial activity. The synthesis of oxime derivatives was performed according to the methodology published by Bednarczyk-Cwynar et al. (2015). They also describe that these oxime derivatives can undergo further syntheses, such as acylation or alkylation. Additionally, these two studies demonstrate that oxime esters in the lupane structure represent a promising pharmacological potential. Information about the synthesis of all tested derivatives, as well as their previously established biological properties and toxicity, is described in our previous research (Černá et al. 2024). It was shown that several oxime derivatives of betulonic and platanic acids exhibited moderate cytotoxicity against human cancer cell lines, while some derivatives demonstrated selective antiviral activity, such as the platanic acid oxime ester (**17**) against HIV-1. These findings suggest that the biological effects of triterpenoid derivatives can vary significantly depending on their specific structure and that selectivity can be achieved (Černá et al. 2024). The observed biological activities of the newly synthesized oxime derivatives are likely influenced by the nature and position of substituents on the triterpenoid backbone. The steric and planar features of these substituents can influence interactions with biological targets, as has been reported for pentacyclic triterpenoids, including betulonic acid derivatives, in structure-activity studies (Lou et al. 2025; Kahnt et al. 2018). From these observations, correlations between structure and biological activity can be inferred, which are crucial for designing derivatives with desired selectivity and efficacy.

The antibacterial activity of the derivatives was evaluated using three methods: disk diffusion, microdilution, and the resazurin assay (Table 4). The differences between the disk diffusion and microdilution results arise from the distinct nature of the methods: disk diffusion reflects compound diffusion in agar, while microdilution measures growth inhibition in liquid culture. Variations in solubility, diffusion, and bacterial growth dynamics can therefore lead to differences in the final results. For the microdilution method, specific growth rates *µ* [1/h] were measured to provide a quantitative assessment of bacterial growth over time, while the growth curves presented in the Supplementary material allowed an estimation of MIC values for the most active derivatives. The combination of these complementary methods provides a comprehensive assessment of compound effectiveness. The results showed that most of our samples exhibited activity primarily against Gram-positive bacterial strains. Growth inhibition was observed against *S. aureus* and *E. faecalis* for most of the derivatives. These findings are consistent with similar results reported by Chue et al. (2011). Haque et al. (2014) reported similar results regarding the lack of activity against Gram-negative bacteria such as *E. coli* and *P. aeruginosa*. The selective activity against Gram-positive bacteria can be explained by structural differences in the cell walls of Gram-positive and Gram-negative bacteria. The outer membrane of Gram-negative bacteria likely impedes the penetration of these triterpenoid derivatives, which explains the minimal inhibition observed for *E. coli* and *P. aeruginosa*.

Focusing on a similar topic, Kazakova et al. (2010) found that among unmodified triterpenoic acids, betulonic acid exhibited the highest antibacterial activity against *S. aureus*. Our results confirm this finding, as unmodified betulonic acid showed better activity compared to unmodified platanic acid. Betulonic acid demonstrated activity against *S. aureus* in both disk diffusion and microdilution tests, with bacterial growth inhibited to a zero specific growth rate. However, in the resazurin assay, betulonic acid showed no significant inhibition. On the other hand, against *E. faecalis*, unmodified betulonic acid exhibited a high percentage of inhibition (93.9% at 500 µmol/L), along with a zero specific growth rate. The higher inhibition of betulonic acid against *E. faecalis* compared to *S. aureus* is consistent with the findings of Haque et al. (2014). They measured the antibacterial activity of betulonic acid at a concentration of 50 µmol/L and found 74.3% inhibition against *E. faecalis* and 51.1% against *S. aureus.* Kazakova et al. (2010) detected the inhibition of *S. aureus* growth caused by betulonic acid at the concentration of 80 mg/mL (corresponding to 175 mmol/L). In our study, the compounds were tested at a lower concentration (500 − 62.5 µmol/L), with similar results. In the study by Poumale et al. (2010), unmodified platanic acid inhibited the growth of *S. aureus* and *E. coli* in the diffusion method at the concentration of 40 mg/mL, which corresponds to 87.2 mmol/L. We obtained slightly different results, as unmodified platanic acid did not show significant inhibition against *S. aureus* ATCC strains. However, we observed strong inhibition against a clinical isolate, *S. aureus* 368.

Our research revealed enhanced antibacterial activity following the modification of triterpenoic acids. Based on our findings, the increased antibacterial activity of several oxime and oxime ester derivatives demonstrates that chemical modification of the triterpenoid scaffold can significantly influence biological efficacy. In particular, derivatives **3**, **7**, **14** and **18** exhibited complete inhibition of growth of *S. aureus* even at concentrations of 125 µmol/L (see **Supplementary material**). Derivative **14** was the only tested compound that inhibited growth of *S. aureus* by 100% at the lowest tested concentration (62.5 µmol/L) (Fig. 1). Derivative **3** showed complete growth inhibition of *E. faecalis* and significantly reduced the specific growth rate and metabolic activity, confirming that modification of betulonic acid via an oxime group enhances activity against selected Gram-positive strains. Another crucial finding is that derivatives **3**, **14** and **18** maintained significant activity against resistant clinical isolates *S. aureus* 368, *S. aureus* 670 or *S. epidermidis* 335, achieving high levels of metabolic inhibition (90–100%). Derivatives **14** and **18** additionally showed approximately 100% growth inhibition of strain *S. epidermidis* 335, highlighting its promising potential against resistant bacteria. These results suggest that the introduction of ester or oxime ester functional groups not only increases antibacterial efficacy but may also overcome certain resistance mechanisms.

Overall, these findings support the hypothesis that functionalization of platanic and betulonic acids can improve antibacterial properties, particularly against Gram-positive bacteria. The most active derivatives (**3**, **7**, **14** and **18**) represent promising candidates for further optimization and development as potential therapeutic agents. Future work should focus on detailed mechanistic studies to elucidate their mode of action.

## Conclusion

We have demonstrated that the structural modification of betulonic and platanic acids represents a promising strategy for the development of new antibacterial agents with potential applications in the treatment of infectious diseases. In our previous work, we successfully designed and synthesized 13 oxime derivatives and four intermediates of these acids. In the present study, several of these derivatives and intermediates exhibited significantly improved antibacterial activity, particularly against Gram-positive bacteria. Notably, complete growth inhibition of *S. aureus* was already observed at reduced concentrations for derivatives **3**, **7** and **18**, whereas derivative **14** achieved 100% inhibition at the lowest tested concentration of 62.5 µmol/L. Derivatives **14** and **18** (500 µmol/L) achieved 90–100% growth inhibition against multiple Gram-positive strains, including resistant clinical isolates such as *S. aureus* 368 and *S. epidermidis* 335. Moreover, derivatives **3**,** 7** and **14** inhibited the growth of *E. faecalis* and exhibited a zero specific growth rate against both Gram-positive bacteria, further confirming its high antibacterial potency. Based on these findings, the betulonic acid oxime (**3**), the betulonic acid oxime ester substituted with pyrazine-2-carboxylic acid (**7**) or 1-adamantylacetic acid (**14**) and the platanic acid acetate intermediate (**18**) should be highlighted as the most promising and selective antibacterial compounds of the studied series. Although the tested derivatives showed no activity against Gram-negative strains, the results indicate that selective structural modifications can substantially enhance antibacterial potency. We also hypothesize that such modifications may influence other pharmacological properties, including antiviral and anticancer potential. Overall, our findings provide a solid foundation for further research, particularly aimed at elucidating the mechanisms underlying these biological effects and optimizing derivatives to achieve desired selectivity and efficacy.

## Supplementary Information

Below is the link to the electronic supplementary material.

Supplementary Material 1 (DOCX 54.5 KB)

## Data Availability

Data will be made available on request.

## Abbreviations

A: Absorbance
CCM: Czech Collection of Microorganisms
CFU: Colony–Forming Units
CIP: ciprofloxacin
CLI: clindamycin
CMP: chloramphenicol
COT: cotrimoxazole
DMSO: dimethyl sulfoxide
ERY: erythromycin
GEN: gentamicin
LNZ: linezolid
McF: McFarland
MHA: Mueller–Hinton Agar
MHB: Mueller–Hinton Broth
MDR: Multidrug–resistant
MRSA: methicillin–resistant *S. aureus*
OXA: oxacillin
TET: tetracycline
WHO: World Health Organization
