# Supplementary material for: Derivatives and intermediates of betulonic and platanic acid as effective antibacterial agents
Source: Folia Microbiol (Praha). 2026 Apr 16;71(5):1473–83. doi: 10.1007/s12223-026-01455-9 (PMC13736308; doi:10.1007/s12223-026-01455-9)
Supplement: Supplementary file 1 [file 12223_2026_1455_MOESM1_ESM.docx]

Growth curves of *S. aureus* after exposure to betulonic acid (**1**) at concentrations of 250 µmol/L, 125 µmol/L and 62.5 µmol/L and incubation at 35 °C for 24 h. The 1% DMSO sample represents the bacterial growth curve as a negative control. The positive control is the bacterial growth curve inhibited by vancomycin at a concentration of 250 µg/mL.

1

Absorbance 625 [nm]

| Time [h] | 250 µmol/L | 125 µmol/L | 62.5 µmol/L | DMSO | Vancomycin |
| --- | --- | --- | --- | --- | --- |
| 1 | 0.162667 | 0.111333 | 0.102667 | 0.103 | 0.0961667 |
| 2 | 0.163 | 0.111 | 0.102667 | 0.104333 | 0.0988333 |
| 3 | 0.162667 | 0.111667 | 0.103667 | 0.107667 | 0.1 |
| 4 | 0.163667 | 0.113 | 0.106 | 0.118444 | 0.100167 |
| 5 | 0.165 | 0.117 | 0.114667 | 0.141 | 0.100167 |
| 6 | 0.170667 | 0.125 | 0.127667 | 0.162889 | 0.100333 |
| 7 | 0.179333 | 0.133667 | 0.137667 | 0.184 | 0.100333 |
| 8 | 0.187667 | 0.139667 | 0.145 | 0.206556 | 0.100167 |
| 9 | 0.194667 | 0.145667 | 0.151667 | 0.229111 | 0.100167 |
| 10 | 0.200333 | 0.15 | 0.158 | 0.255333 | 0.100167 |
| 11 | 0.205 | 0.153667 | 0.164667 | 0.285333 | 0.1 |
| 12 | 0.208333 | 0.157 | 0.171667 | 0.323111 | 0.100167 |
| 13 | 0.211667 | 0.161 | 0.179333 | 0.372222 | 0.100167 |
| 14 | 0.214667 | 0.164333 | 0.189333 | 0.445111 | 0.100333 |
| 15 | 0.216667 | 0.167667 | 0.201333 | 0.532778 | 0.100167 |
| 16 | 0.219 | 0.172 | 0.214667 | 0.631778 | 0.100333 |
| 17 | 0.220667 | 0.176333 | 0.231667 | 0.729444 | 0.100833 |
| 18 | 0.222333 | 0.182 | 0.249667 | 0.808333 | 0.100833 |
| 19 | 0.224 | 0.187667 | 0.27 | 0.878 | 0.100833 |
| 20 | 0.224 | 0.195 | 0.295667 | 0.948667 | 0.100833 |
| 21 | 0.224333 | 0.202 | 0.325333 | 1.01022 | 0.100667 |
| 22 | 0.225333 | 0.211333 | 0.365 | 1.043 | 0.100667 |
| 23 | 0.226 | 0.221667 | 0.403333 | 1.07944 | 0.100833 |
| 24 | 0.226667 | 0.233667 | 0.461667 | 1.11089 | 0.101 |

Growth curves of *S. aureus* after exposure to derivative **3** at concentrations of 250 µmol/L, 125 µmol/L and
62.5 µmol/L and incubation at 35 °C for 24 h. The 1% DMSO sample represents the bacterial growth curve as a negative control. The positive control is the bacterial growth curve inhibited by vancomycin at a concentration of 250 µg/mL.

3

Absorbance 625 [nm]

| Time [h] | 250 µmol/L | 125 µmol/L | 62.5 µmol/L | DMSO | Vancomycin |
| --- | --- | --- | --- | --- | --- |
| 1 | 0.114333 | 0.103667 | 0.0963333 | 0.103 | 0.0961667 |
| 2 | 0.114 | 0.104333 | 0.0966667 | 0.104333 | 0.0988333 |
| 3 | 0.114667 | 0.104667 | 0.097 | 0.107667 | 0.1 |
| 4 | 0.115 | 0.105 | 0.0976667 | 0.118444 | 0.100167 |
| 5 | 0.115667 | 0.106 | 0.1 | 0.141 | 0.100167 |
| 6 | 0.116 | 0.107667 | 0.105333 | 0.162889 | 0.100333 |
| 7 | 0.116667 | 0.111667 | 0.114333 | 0.184 | 0.100333 |
| 8 | 0.117667 | 0.117667 | 0.124 | 0.206556 | 0.100167 |
| 9 | 0.119 | 0.125667 | 0.134 | 0.229111 | 0.100167 |
| 10 | 0.121667 | 0.134333 | 0.143 | 0.255333 | 0.100167 |
| 11 | 0.125667 | 0.143 | 0.152 | 0.285333 | 0.1 |
| 12 | 0.130333 | 0.150667 | 0.161 | 0.323111 | 0.100167 |
| 13 | 0.136333 | 0.157333 | 0.170667 | 0.372222 | 0.100167 |
| 14 | 0.142667 | 0.164 | 0.179667 | 0.445111 | 0.100333 |
| 15 | 0.149333 | 0.170333 | 0.19 | 0.532778 | 0.100167 |
| 16 | 0.155333 | 0.176333 | 0.200333 | 0.631778 | 0.100333 |
| 17 | 0.161667 | 0.183 | 0.212333 | 0.729444 | 0.100833 |
| 18 | 0.167667 | 0.189 | 0.226667 | 0.808333 | 0.100833 |
| 19 | 0.174 | 0.195333 | 0.242667 | 0.878 | 0.100833 |
| 20 | 0.180667 | 0.201 | 0.259667 | 0.948667 | 0.100833 |
| 21 | 0.187 | 0.206667 | 0.287 | 1.01022 | 0.100667 |
| 22 | 0.192333 | 0.212333 | 0.314 | 1.043 | 0.100667 |
| 23 | 0.197667 | 0.218 | 0.335667 | 1.07944 | 0.100833 |
| 24 | 0.203 | 0.223333 | 0.365333 | 1.11089 | 0.101 |

Growth curves of *S. aureus* after exposure to derivative **7** at concentrations of 250 µmol/L, 125 µmol/L and
62.5 µmol/L and incubation at 35 °C for 24 h. The 1% DMSO sample represents the bacterial growth curve as a negative control. The positive control is the bacterial growth curve inhibited by vancomycin at a concentration of 250 µg/mL.

7

Absorbance 625 [nm]

| Time [h] | 250 µmol/L | 125 µmol/L | 62.5 µmol/L | DMSO | Vancomycin |
| --- | --- | --- | --- | --- | --- |
| 1 | 0.137333 | 0.114333 | 0.107667 | 0.103 | 0.0961667 |
| 2 | 0.133 | 0.114 | 0.106667 | 0.104333 | 0.0988333 |
| 3 | 0.13 | 0.114333 | 0.106667 | 0.107667 | 0.1 |
| 4 | 0.128 | 0.113333 | 0.107667 | 0.118444 | 0.100167 |
| 5 | 0.126667 | 0.114 | 0.108333 | 0.141 | 0.100167 |
| 6 | 0.126333 | 0.115 | 0.110667 | 0.162889 | 0.100333 |
| 7 | 0.125333 | 0.117 | 0.114 | 0.184 | 0.100333 |
| 8 | 0.125333 | 0.119667 | 0.119 | 0.206556 | 0.100167 |
| 9 | 0.125333 | 0.122333 | 0.124667 | 0.229111 | 0.100167 |
| 10 | 0.125667 | 0.126333 | 0.132 | 0.255333 | 0.100167 |
| 11 | 0.127 | 0.131333 | 0.138333 | 0.285333 | 0.1 |
| 12 | 0.128 | 0.137333 | 0.145333 | 0.323111 | 0.100167 |
| 13 | 0.131 | 0.144667 | 0.153333 | 0.372222 | 0.100167 |
| 14 | 0.132667 | 0.152 | 0.163333 | 0.445111 | 0.100333 |
| 15 | 0.137667 | 0.159333 | 0.173667 | 0.532778 | 0.100167 |
| 16 | 0.143 | 0.167333 | 0.184 | 0.631778 | 0.100333 |
| 17 | 0.148333 | 0.175 | 0.195 | 0.729444 | 0.100833 |
| 18 | 0.154 | 0.183333 | 0.207 | 0.808333 | 0.100833 |
| 19 | 0.16 | 0.191 | 0.218667 | 0.878 | 0.100833 |
| 20 | 0.166 | 0.198667 | 0.230667 | 0.948667 | 0.100833 |
| 21 | 0.172 | 0.206667 | 0.243667 | 1.01022 | 0.100667 |
| 22 | 0.177333 | 0.215 | 0.258 | 1.043 | 0.100667 |
| 23 | 0.183333 | 0.223 | 0.274 | 1.07944 | 0.100833 |
| 24 | 0.19 | 0.232 | 0.289333 | 1.11089 | 0.101 |

Growth curves of *S. aureus* after exposure to derivative **18** at concentrations of 250 µmol/L, 125 µmol/L and
62.5 µmol/L and incubation at 35 °C for 24 h. The 1% DMSO sample represents the bacterial growth curve as a negative control. The positive control is the bacterial growth curve inhibited by vancomycin at a concentration of 250 µg/mL.

18

Absorbance 625 [nm]

| Time [h] | 250 µmol/L | 125 µmol/L | 62.5 µmol/L | DMSO | Vancomycin |
| --- | --- | --- | --- | --- | --- |
| 1 | 0.117 | 0.0996667 | 0.0956667 | 0.103 | 0.0961667 |
| 2 | 0.117333 | 0.0996667 | 0.096 | 0.104333 | 0.0988333 |
| 3 | 0.118 | 0.100667 | 0.097 | 0.107667 | 0.1 |
| 4 | 0.119 | 0.101 | 0.0986667 | 0.118444 | 0.100167 |
| 5 | 0.119333 | 0.101667 | 0.102667 | 0.141 | 0.100167 |
| 6 | 0.12 | 0.102333 | 0.109667 | 0.162889 | 0.100333 |
| 7 | 0.122 | 0.102667 | 0.125667 | 0.184 | 0.100333 |
| 8 | 0.122667 | 0.103333 | 0.140667 | 0.206556 | 0.100167 |
| 9 | 0.124667 | 0.104 | 0.173333 | 0.229111 | 0.100167 |
| 10 | 0.129333 | 0.104667 | 0.232333 | 0.255333 | 0.100167 |
| 11 | 0.131667 | 0.105 | 0.3 | 0.285333 | 0.1 |
| 12 | 0.134667 | 0.105667 | 0.370667 | 0.323111 | 0.100167 |
| 13 | 0.138667 | 0.106667 | 0.430667 | 0.372222 | 0.100167 |
| 14 | 0.140333 | 0.108333 | 0.505667 | 0.445111 | 0.100333 |
| 15 | 0.143 | 0.109 | 0.567667 | 0.532778 | 0.100167 |
| 16 | 0.143333 | 0.110333 | 0.631333 | 0.631778 | 0.100333 |
| 17 | 0.146667 | 0.109667 | 0.692667 | 0.729444 | 0.100833 |
| 18 | 0.150667 | 0.111667 | 0.750667 | 0.808333 | 0.100833 |
| 19 | 0.150667 | 0.111667 | 0.794 | 0.878 | 0.100833 |
| 20 | 0.152 | 0.111333 | 0.851 | 0.948667 | 0.100833 |
| 21 | 0.153667 | 0.113 | 0.898 | 1.01022 | 0.100667 |
| 22 | 0.156667 | 0.114 | 0.925333 | 1.043 | 0.100667 |
| 23 | 0.160333 | 0.115333 | 0.957 | 1.07944 | 0.100833 |
| 24 | 0.158667 | 0.117667 | 0.986333 | 1.11089 | 0.101 |

Measurement uncertainty (±15%) associated with the antibacterial activity results of the tested triterpenoid derivatives.

| **Tested substance** | **± 15% Uncertainty** | | | | | | | | |
| --- | --- | --- | --- | --- | --- | --- | --- | --- | --- |
|  | ***S. aureus*** | | ***E. faecalis*** | | ***E. coli*** | | ***P. aeruginosa*** | |  |
|  | ***µ*** × **10^-2^ [1/h]** | ***I***  **[%]** | ***µ*** × **10^-2^ [1/h]** | ***I***  **[%]** | ***µ*** × **10^-2^ [1/h]** | ***I***  **[%]** | ***µ*** × **10^-2^ [1/h]** | ***I***  **[%]** |  |
| 1 % DMSO | ± 0.61 | ± 0.00 | ± 0.50 | ± 0.00 | ± 0.91 | ± 0.00 | ± 1.16 | ± 0.00 |  |
| Vancomycin | ± 0.00 | ± 15.00 | ± 0.00 | ± 15.00 | - | - | - | - |  |
| Kanamycin | - | - | - | - | ± 0.00 | ± 15.00 | ± 0.00 | ± 15.00 |  |
| **1** | ± 0.00 | - | ± 0.00 | ± 14.09 | ± 0.84 | ± 3.48 | ± 0.27 | - |  |
| **2** | ± 0.12 | - | ± 0.25 | ± 0.81 | ± 1.13 | ± 0.39 | ± 0.55 | - |  |
| **3** | ± 0.00 | - | ± 0.00 | ± 15.00 | ± 0.84 | ± 0.90 | ± 0.38 | - |  |
| **4** | ± 0.13 | - | ± 0.21 | ± 12.99 | ± 0.59 | - | ± 0.48 | - |  |
| **5** | ± 0.30 | ± 2.04 | ± 0.26 | ± 8.36 | ± 1.06 | ± 0.14 | ± 0.55 | - |  |
| **6** | ± 0.34 | - | ± 0.00 | ± 2.85 | ± 0.44 | - | ± 0.54 | - |  |
| **7** | ± 0.00 | ± 0.48 | ± 0.00 | ± 9.06 | ± 0.45 | - | ± 0.47 | - |  |
| **8** | ± 0.14 | ± 1.58 | ± 0.11 | ± 9.15 | ± 0.42 | ± 0.14 | ± 0.32 | - |  |
| **9** | ± 0.35 | - | ± 0.99 | - | ± 0.77 | - | ± 0.52 | ± 3.42 |  |
| **10** | ± 1.08 | - | ± 1.42 | - | ± 0.57 | - | ± 0.60 | - |  |
| **11** | ± 0.82 | ± 1.82 | ± 0.42 | ± 0.98 | ± 0.27 | - | ± 0.73 | - |  |
| **12** | ± 0.20 | ± 5.54 | ± 0.19 | ± 2.82 | ± 0.33 | ± 2.51 | ± 0.88 | - |  |
| **13** | ± 0.42 | - | ± 0.74 | ± 1.89 | ± 0.64 | - | ± 0.39 | ± 2.49 |  |
| **14** | ± 0.00 | ± 9.69 | ± 0.00 | ± 10.58 | ± 0.54 | - | ± 0.45 | ± 4.89 |  |
| **15** | ± 0.34 | - | ± 0.15 | ± 1.49 | ± 0.83 | - | ± 0.77 | - |  |
| **16** | ± 0.17 | - | ± 0.16 | ± 10.40 | ± 0.62 | - | ± 0.35 | - |  |
| **17** | ± 0.15 | ± 6.45 | ± 0.23 | ± 11.18 | ± 0.81 | ± 1.95 | ± 0.78 | - |  |
| **18** | ± 0.00 | ± 14.43 | ± 0.60 | ± 12.45 | ± 1.95 | ± 4.61 | ± 0.47 | ± 3.66 |  |
| **19** | ± 0.14 | - | ± 0.22 | ± 3.50 | ± 0.90 | ± 0.62 | ± 0.59 | - |  |

Note: *µ* × 10^-2^ [1/h] represents the specific growth rate and I [%] represents the inhibition of metabolic activity.
